# Supplementary figures and images for: Spatiotemporal structure of SARS-CoV-2 mutational frequencies in wastewater samples from Ontario
Source: PLoS One. 2025 Oct 16;20(10):e0333945. doi: 10.1371/journal.pone.0333945 (PMC12530563; doi:10.1371/journal.pone.0333945)

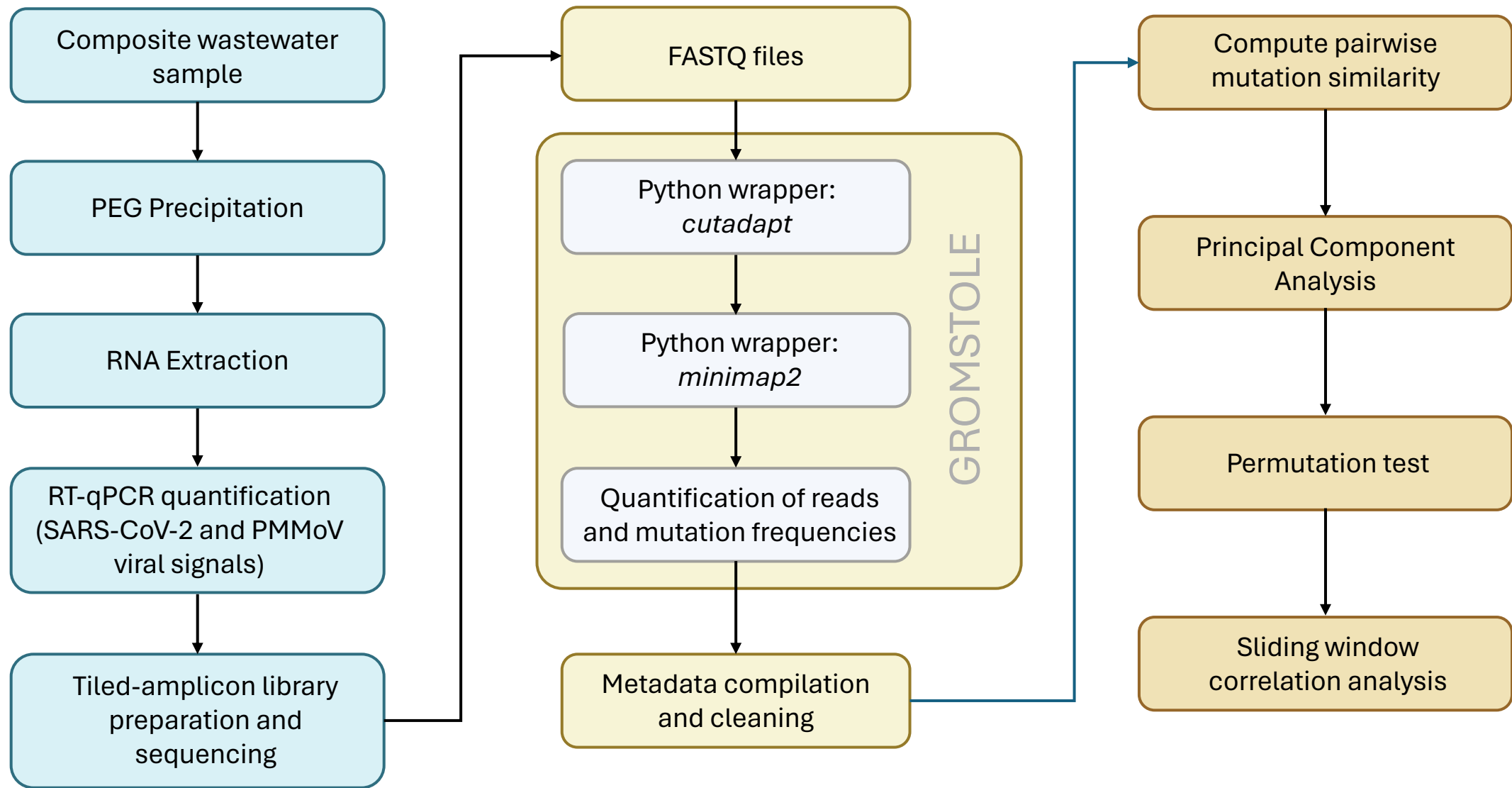

Supplement: S1 Fig — (PDF) [file pone.0333945.s001.pdf]

Month of sampling

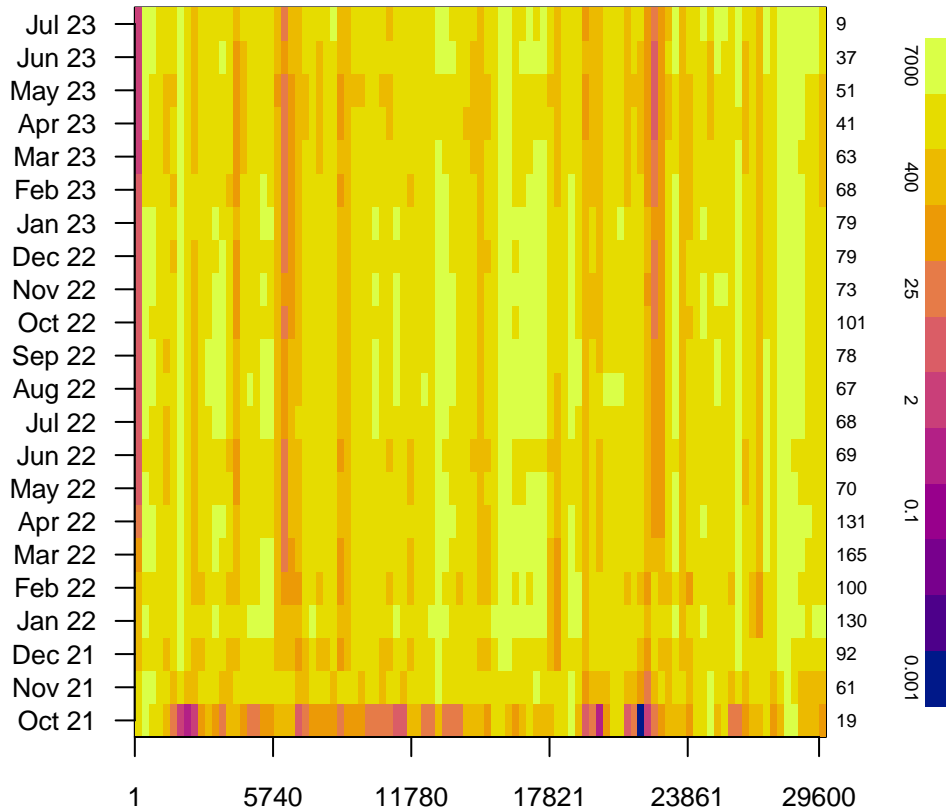

Genome coordinates

Supplement: S2 Fig — The average log-transformed read depths are mapped to a colour gradient derived from the accessible ‘Plasma’ palette in R — a legend is provided on the right-hand side of the plot. The number of samples per month is indicated along the right margin of the plot. (PDF) [file pone.0333945.s002.pdf]

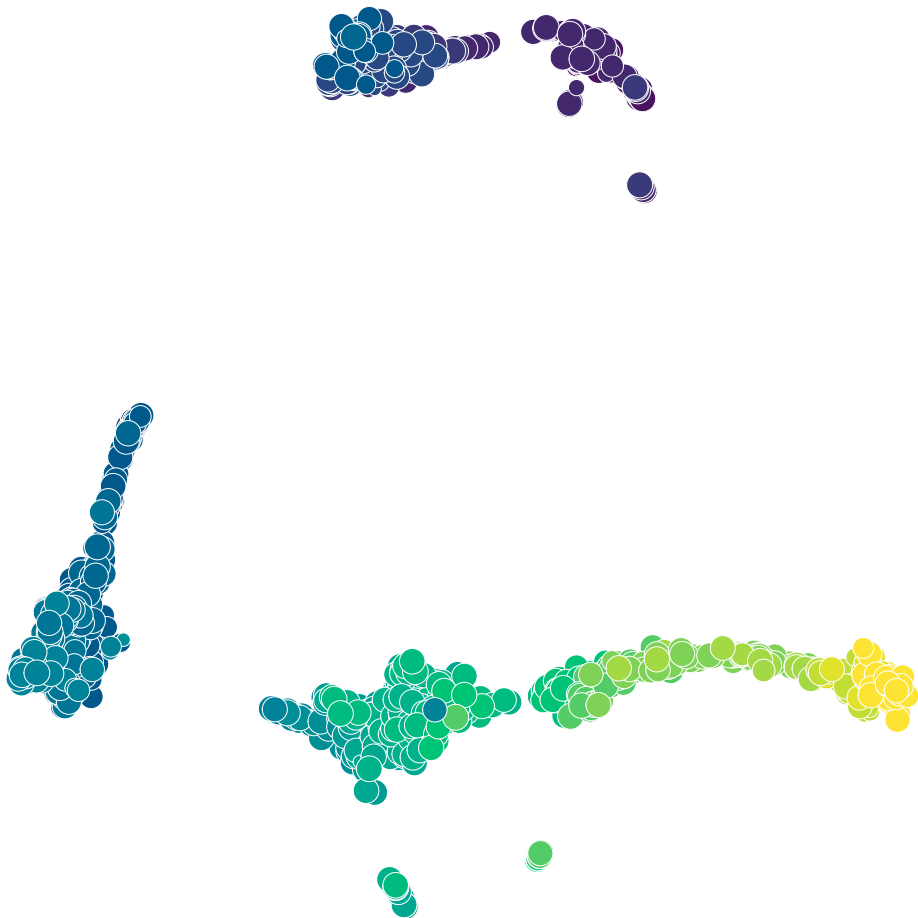

Supplement: S3 Fig — Similarly to Fig 7, each point represents a sample, with its area scaled in proportion to coverage (number of nucleotide sites with a minimum depth of 100 reads), and coloured by month of sample collection. (PDF) [file pone.0333945.s003.pdf]

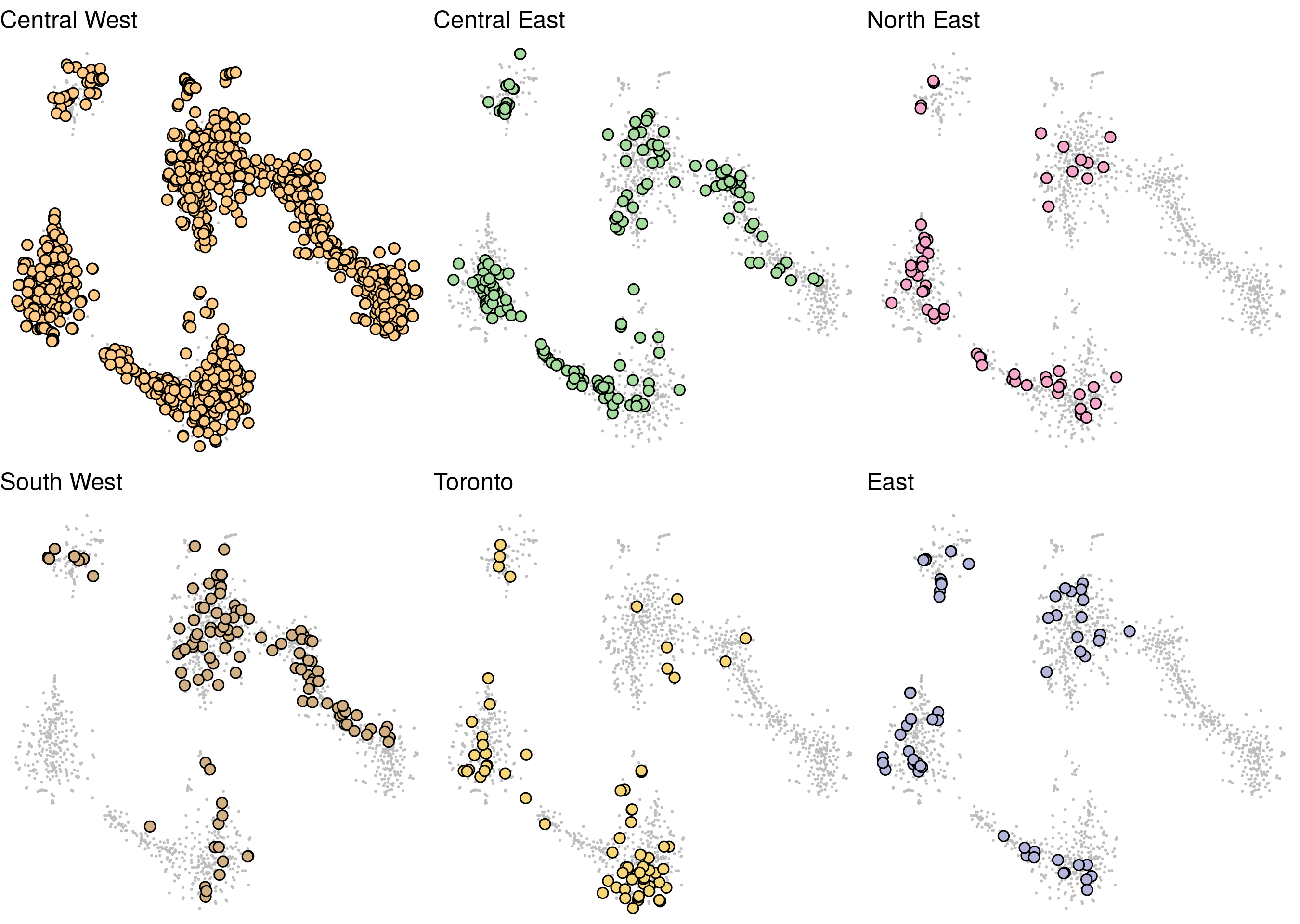

Supplement: S4 Fig — Geographic boundaries of health regions are highlighted in Fig 1. Samples from other regions are represented by small grey points. (PNG) [file pone.0333945.s004.png]
