# Supplementary material for: Spatiotemporal structure of SARS-CoV-2 mutational frequencies in wastewater samples from Ontario
Source: PLoS One. 2025 Oct 16;20(10):e0333945. doi: 10.1371/journal.pone.0333945 (PMC12530563; doi:10.1371/journal.pone.0333945)
Supplement: Attachment — Submitted filename: ResponseToReviewers.pdf [file pone.0333945.s005.pdf]

Schulich
MEDICINE & DENTISTRY

Pathology and
Laboratory Medicine

September 4, 2025

Dear colleagues,

On behalf of my co-authors, I am submitting a revised version of our manuscript '*Spatiotemporal structure of SARS-CoV-2 mutational frequencies in wastewater samples from Ontario*' (PONE-D-25-23235) for your consideration as a Research Article.

We are grateful to both reviewers for their thorough and insightful assessment of our previous manuscript, and we thank the editor for this opportunity to submit a revision. In the following section, we provide an itemized list of the reviewers' comments, as well as our response to each point, with references to the revised manuscript where applicable.

Reviewer #1

1. *"Congratulations to this very nice (and interesting) manuscript. It is clearly written, nicely explained and easy to follow."*

   Thank you very much for your supportive feedback!

2. *"p. 3, lines 3-11: Suggest to move to methods section as study area sub-section."*

   We have moved the indicated text to the Methods section as suggested by the reviewer.

3. *"p. 4, lines 20-23: May this have an impact on the number of mutations detected?"*

   The reviewer is referring to the use of different versions of the ARTIC sequencing protocol (primer sets) over the course of the study. The hypothesis implied by the reviewer's comment is that changing protocols affected our ability to detect mutations. This is difficult to test because the numbers of fixed substitutions and polymorphic sites have also changed over time. In other words, the effect of sequencing methods and the underlying genetic variation are confounded. However, we note that the major version change between ARTIC v3 and v4 occurred within the first few months of the 22-month study period, followed by a switch to v4.1 only three months afterwards. Although the switch from v3 to v4 appears to be associated with a shift in the distribution of read depths among tiles between the first and second months of sampling (Supplementary Figure S2), the overall distribution was quite consistent over the remaining time. Moreover, we note that neglecting to update sequencing protocols would also have an impact on the detection of mutations, as revised primer sequences were designed to accommodate the ongoing evolution of the SARS-CoV-2 genome binding sites. We have expanded on our discussion of this potential issue in the Discussion section of the revised manuscript (see point 8 below).

4. *"p. 5, Figure 1: Please add the number to the legend to show the magnitude of the number of samples, e.g. is a small circle one sample or ten or hundred?"*

   This is an excellent suggestion. We have amended this figure with reference circles labeled with the corresponding sample sizes as a visual aid for the reader. We have reproduced the revised figure at a smaller scale here for your convenience:

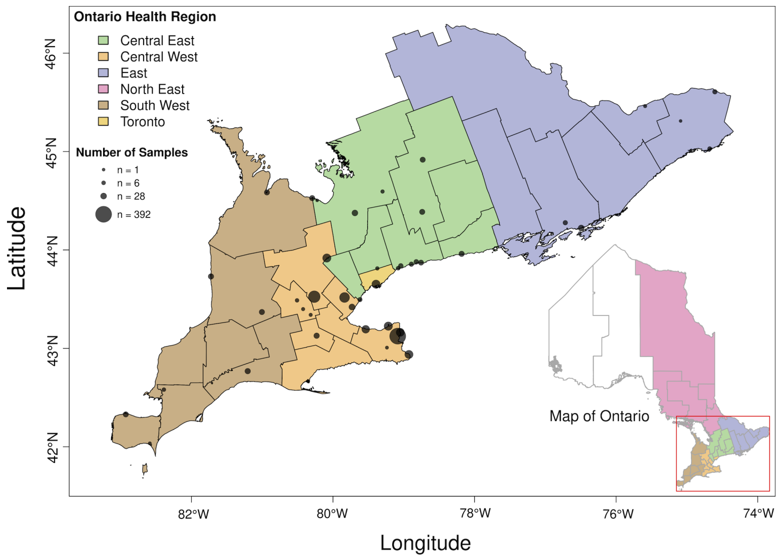

5. *"p. 7, line 1: "This normalization also conferred some robustness to variation in read coverage among samples...", Please discuss this further as it has an impact on your analysis, i.e. deriving spatial or temporal differences when in reality read coverages were different, i.e. not the same mutations were covered."*

   We have expanded on our discussion of this aspect of the cosine similarity measure, *i.e.*, that is confers some robustness to differences in read coverage, with reference to the literature:

   > This normalization also conferred some robustness to variation in read coverage among samples. To understand this property, consider the set of frequencies in a sample, $f(x)$, as a vector in a high-dimensional space where every dimension represents a different mutation. The quantity $\langle x, y\rangle'$ represents the angle between two vectors rooted at the origin. Although a loss of coverage collapses some dimensions in this enormous space, the angle between vectors is largely preserved. Hence, cosine similarity has used as a basis for imputing missing data (Rashid *et al.* 2020) and computing similarity matrices on incomplete data (Yu *et al.* 2023). It is also a popular choice in the field of text mining due to its robustness to variation in document lengths (Huang *et al.* 2008).

6. *"General observation: Figures often placed before their call-outs in the text."*

   We apologize for this inconvenience. This will be addressed in our revision, as we have removed the embedded images from the manuscript, and relocated figure legends to immediately follow the paragraph containing the first in-text citation as instructed by PLOS ONE staff.

7. *"p. 20, lines 6-7: Have you considered examining defining mutations for a variant to see whether your approach would be comparable to other data sources in terms of the time component?"*

   We compared the frequencies of mutations associated with the introduction of major variants to the region over time, as illustrated in Figure 6. The introduction of these variants was estimated from other data sources — primarily the estimated frequencies of variants based on full-length genome sequencing of individual samples (*e.g.*, CoVaRR-Net Duotang, https://covarr-net.github.io/duotang/duotang.html). We had not considered comparing these mutational trends to additional data sources, as we are not aware of other sources (*i.e.*, other than genomic sequencing) that could provide a regional breakdown of lineage- or variant-specific frequencies over time.

8. *"p. 20, lines 14-15: How do you ensure that this is really due to temporal differences in the occurrence rather than differences in coverages?"*

   Thank you for raising this point. Although we cannot entirely rule out the possibility that the observed temporal structure was driven by changes in sequencing coverage, our results are not consistent with differences in coverage as a primary driver. First, the distribution of mean read depths among amplicon 'tiles' was generally consistent over time (Supplementary Figure S2), with the exception of the first few months where the ARTIC sequencing protocol was updated from v3 to v4. In addition, the frequencies of mutations associated with major variants were broadly consistent with the introduction of those variants into the region over time (Figure 6). We have expanded on this point in the Discussion to clarify this point:

   > Another potential issue is that we transitioned between three different versions of the ARTIC protocol (V3, V4 and V4.1) over the study period to compensate for effect of mutations on the amplification of specific 'tiles'. It is possible that updating the primer sets may have influenced the number of mutations detected in our data. On the other hand, neglecting to update the protocol would have induced a sampling bias, as ongoing evolution of the SARS-CoV-2 genome would have reduced the efficiency of PCR amplification over time (Davis *et al.* 2021). It is difficult to assess the impact of these changes on our ability to detect mutations, because the numbers of fixed substitutions and polymorphic sites would have also changed over time. In other words, the effects of sequencing methodologies and the underlying genetic variation are confounded. However, we observed that the distribution of average read depths across tiles was generally consistent over time, with the exception of the first month in our study period (October 2021; S2 Fig.). Furthermore, we employed analytical methods (*i.e.*, cosine similarity) that are less sensitive to variation in coverage, as opposed to lineage-based methods that rely on the presence or absence of specific mutations.

9. *"p. 20, lines 16-17: Please consider: statistically significant yes, but also practically relevant?"*

   This is a fair point. The fact that spatial structure was not evident in our data visualizations implies that our results are not epidemiologically significant. We have amended this section to make a clearer distinction between these different kinds of significance:

   > In addition, our permutation tests indicated that there was statistically significant differentiation between samples from proximal versus distant locations that were collected at similar points in time. However, the fact that we could not readily discern spatial structure among samples using data visualizations (*e.g.*, Figure 7) implies that this result may have limited epidemiological significance.

10. *"p. 21, lines 9-10: How did you derive this lower limit of detection for spatial structure, as there are studies showing differences between geographical units on a much smaller spatial scale?"*

    This is an excellent point. We have revised this section with reference to previous studies that have found differences at smaller spatial scales (see also points 12 and 13 below):

    > This geographic scale may be near the lower limit of detection for spatial structure in wastewater genomic data. On the other hand, other studies have been able to differentiate between locations at a similar geographic scale. For example, Agrawal *et al.* detected mutations characteristic of the Omicron base lineage B.1.1.529 in wastewater

samples weeks before the first clinical case was confirmed by genomic sequencing, and the majority of these mutations were detected in wastewater samples from the Frankfurt Airport instead of the surrounding Frankfurt city area. Subsequent studies also showed that analyzing wastewater in sub-sewer sheds of German metropolitan areas can predict the arrival of a dominant variant (Schmiege *et al.* 2023). In addition, Rios *et al.* (2021) showed this type of analysis can provide information on the expected spatial scale of SARS-CoV-2 community spread at the resolution of neighborhoods within a city.

11. *"p. 21, lines 16-17: Your study focused on single mutations instead of lineage-assignment, for which you highlighted the shortcomings, so why bring back this argument in the discussion?"*

    We apologize for the confusion. We have reworded this sentence to avoid reference to the process of lineage assignment:

    > Identifying differences in the mutational composition of SARS-CoV-2 wastewater samples in relation to their location can reveal important information about how viruses spread from their initial point(s) of introduction (Karthikeyan *et al.* 2022).

12. *"p. 21, lines 17-21: Please provide an example for residential areas, such as Schmiege, Kraiseburd et al.: https://doi.org/10.1016/j.scitotenv.2023.165458"*

    Thank you for providing this example. We have incorporated a reference to Schmiege *et al.* into this section (see point 10 above).

13. *"p. 21, lines 21-22: Please provide an example for this statement, such as Rios et al.: https://doi.org/10.1016/j.lanepe.2021.100202"*

    Thank you for raising this issue. We have added an example, including the suggested reference, in support of this statement in the revised version (see point 10 above).

Reviewer #2

1. *"The authors present a large study of SARS-CoV-2 sequencing of wastewater samples from the Ontario region of Canada. They focus on the nucleotide mutations rather than lineage deconvolution. I found the paper to be interesting, well formatted, and ready for publication. I have no suggestions or questions."*

   We are very grateful for the reviewer's generous commendations.

Sincerely,

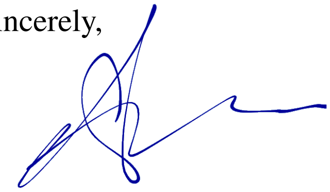

Art F. Y. Poon, MSc PhD
Professor and Chair of Research-Based Graduate Education

Department of Pathology and Laboratory Medicine
Schulich School of Medicine & Dentistry, Western University
Dental Sciences Building, Room 4144, London ON, Canada N6A 5C1
t: 519-661-2111 ext. 87978 e: apoon42@uwo.ca

Western
